# Utility based approach in individualized optimal dose selection using machine learning methods

**DOI:** 10.1002/sim.9396

**Published:** 2022-03-28

**Authors:** Pin Li, Jeremy M. G. Taylor, Philip S. Boonstra, Theodore S. Lawrence, Matthew J. Schipper

**Affiliations:** ^1^ Department of Biostatistics University of Michigan Ann Arbor Michigan USA; ^2^ Department of Radiation Oncology University of Michigan Ann Arbor Michigan USA

**Keywords:** Gaussian process, random forest, utility matrix

## Abstract

The goal in personalized medicine is to individualize treatment using patient characteristics and improve health outcomes. Selection of optimal dose must balance the effect of dose on both treatment efficacy and toxicity outcomes. We consider a setting with one binary efficacy and one binary toxicity outcome. The goal is to find the optimal dose for each patient using clinical features and biomarkers from available dataset. We propose to use flexible machine learning methods such as random forest and Gaussian process models to build models for efficacy and toxicity depending on dose and biomarkers. A copula is used to model the joint distribution of the two outcomes and the estimates are constrained to have non‐decreasing dose‐efficacy and dose‐toxicity relationships. Numerical utilities are elicited from clinicians for each potential bivariate outcome. For each patient, the optimal dose is chosen to maximize the posterior mean of the utility function. We also propose alternative approaches to optimal dose selection by adding additional toxicity based constraints and an approach taking into account the uncertainty in the estimation of the utility function. The proposed methods are evaluated in a simulation study to compare expected utility outcomes under various estimated optimal dose rules. Gaussian process models tended to have better performance than random forest. Enforcing monotonicity during modeling provided small benefits. Whether and how, correlation between efficacy and toxicity, was modeled, had little effect on performance. The proposed methods are illustrated with a study of patients with liver cancer treated with stereotactic body radiation therapy.

## 1 INTRODUCTION

Precision medicine is an approach for the treatment of a disease that takes into account individual variability. In many situations, the clinical decision is whether to give a particular treatment or a standard treatment to the patient, and the statistical goal is to identify the subgroup of patients likely to derive benefit from the treatment compared to standard or population based treatment. In other settings, the treatment choice is not binary and in particular consists of choosing the value of a continuous variable, such as the dose of radiation or a drug. The goal in this setting is to find the optimal dose for each patient to maximize the benefit of treatment. With the development of biomarkers, the goal can be achieved by evaluating how the biomarkers moderate the treatment effect on the outcome or outcomes. In statistical terms moderation of the treatment effect can be formulated as the interaction between the biomarkers and dose in a model.

In this article we consider the setting where there is an existing dataset from previously treated patients that contains treatment dose, observed efficacy and toxicity outcomes and covariates possibly including biomarkers. The available dataset could be from several clinical trials, cohort study, or real‐world evidence studies for which both the sample size is relatively large and there is variation in the dose. The statistical goal is to analyze the data to learn an individualized dosing rule giving an optimal dose as a function of patient level covariates.

Supervised learning in the form of regression (for continuous outputs) and classification (for discrete outputs) is an important constituent of statistics and machine learning. Widely used parametric models such as linear regression and logistic regression are simple, but can suffer from model mis‐specification. Concerns about mis‐specification can be reduced by including extra features, such as interactions and splines, or there are many non‐parametric methods that can be used instead. Because of their greater flexibility, there is less concern about model mis‐specification, but also increased potential for overfitting. Decision trees such as Classification and Regression Trees (CART) and random forest, built upon CART, are easy to understand and have been used in determining the optimal treatment.^1^ Their tree structure handles the interaction well but also increases the potential for overfitting. Methods based on kernel machines are also flexible and have good performance with high‐dimensional data. Examples of these are support vector machines as used in the outcome weighted learning (OWL)^2^ method, and Gaussian process.^3^ Modern Bayesian semiparametric and non‐parametric models also provide posterior uncertainty quantification which can be used to provide uncertainty estimates of patient specific treatment decisions. An example of this is using Bayesian Additive Regression Trees (BART) to provide individual treatment rules (ITR). This approach provides the uncertainty of the outcome associated with the optimal ITR.^4^

In early phase studies in oncology, it is typical to describe the patient outcome in terms of both toxicity (T) such as adverse events and efficacy (E) such as overall response or progression free during specified timeframe. The benefit for each patient can be defined based on combining these two binary outcomes. By maximizing the potential benefit for each patient, the individualized optimal treatment or dose will be selected. Several strategies have been proposed for identifying an optimal treatment or dose based on the trade‐off between efficacy and toxicity. To achieve maximum efficacy with tolerable toxicity, a utility function can be used as a weighted difference of probabilities of efficacy and toxicity.^5^ More generally, a utility matrix is elicited from clinicians by assigning numerical utilities to each possible efficacy and toxicity outcome pair, which allows different preferences for different outcomes and then the utility function, defined as the expected value of the utility, at different dose levels are compared.^6^ Contours characterizing the trade‐off between E and T is an alternative and flexible approach. For this, the set of pairs for the probability of E and the probability of T on the same contour are equally desirable and the dose that maximizes the desirability is selected.^7^

In oncology and other disease settings, it is frequently reasonable to assume that increasing dose leads to increased toxicity and efficacy. However, the increase may not be strictly monotone over the whole range of possible doses. For example, FDA guidance on cellular and gene therapy products mentions that indicators of potential benefit appear to plateau above a certain dose.^8^ Thus, we will consider constraining the estimated dose‐efficacy and dose‐toxicity relationship to be non‐decreasing for all patients. The effect of this should be to reduce the estimation noise, improve efficiency and hence yield more reliable results.

In previous work we built separate models for E and T and did not explicitly consider correlation between E and T.^5^ In this article we relax the assumption of independence and use flexible machine learning methods to build the models for E and T using dose and biomarkers, which differs from the previously developed parametric models. Furthermore, in this article, we define optimal dose as the dose value that maximizes the expected utility where utility values are specified in matrix form for the 4 possible bivariate binary outcomes instead of utility functions, which are equivalent to restricted utility matrices with only a single degree of freedom (ie, one value to specify). In Section 2, we consider alternative uses of the utility function to select an optimal dose, including the addition of constraints with clinical motivation as well as the uncertainty of the utility function. In Section 3, we propose the use of random forest and Gaussian process models to build marginal models and link them with a Gaussian copula. We also build models on joint probabilities and use pool adjacent violators algorithm (PAVA) isotonic transformation to give a non‐decreasing dose‐efficacy and dose‐toxicity relationship. A simulation study to compare different model building and optimal dose finding methods under several scenarios is summarized in Section 4. In Section 5, we illustrate the proposed methods in a dataset of patients with liver cancer treated with stereotactic body radiation therapy (SBRT). We close with discussion in Section 6.

## 2 OPTIMAL DOSE SELECTION

### 2.1 Utility function and matrix

We assume the available data, ${𝒟}_{i}=(x_{i},d_{i},E_{i},T_{i}),i=1,\ldots ,N$, comprises N independent and identically distributed copies of (x,d,E,T), where x is a Q‐dimensional vector of subject‐specific features, d∈[−1,1] denotes continuous dose of treatment, E is the binary efficacy outcome, and T is the binary toxicity outcome. Denote $p_{E}(d,x)=\mathrm{Pr}(E=1|d,x)$ and $p_{T}(d,x)=\mathrm{Pr}(T=1|d,x)$ as the marginal probabilities for the efficacy and toxicity outcomes. One way to combine the efficacy and toxicity outcomes is through a utility function such as $U(p(d,x),\theta )=p_{E}(d,x)-\theta p_{T}(d,x)$ with tradeoff parameter θ>0, either pre‐specified by physicians or calculated as a tuning parameter to meet a pre‐specified level of toxicity in the population.^5^ Alternatively, a unique utility value can also be specified for each possible bi‐variate patient outcome (E,T) in a utility matrix,^6^ as shown in Table 1. We assign $U_{10}$ the highest value to the best possible outcome (efficacy and no toxicity), and $U_{01}$ the lowest value to the worse possible outcome (toxicity and no efficacy). $U_{00}$ and $U_{11}$ have values in between and can be larger or smaller or equal to each other. Denote the joint probability of E and T given d and x as $p_{ET}(d,x)$ for E,T in {0,1}. We define a utility function as the expectation of this utility matrix with respect to the joint probability of E and T at dose d and covariates x. The goal is then to maximize the utility function, or the expectation of the utility matrix for fixed x at a grid of possible dose values.

**TABLE 1:** Utility matrix

	E=0	E=1
T=0	$U_{00}$	$U_{10}$
T=1	$U_{01}$	$U_{11}$

The utility function above, $U(p(d,x),\theta )=p_{E}(d,x)-\theta p_{T}(d,x)$, corresponds to one with $\left(U_{00},U_{10},U_{01},U_{11})=(0,1,-\theta ,1-\theta \right)$. Without loss of generality, we can assign the highest utility as 1 and lowest utility as 0.^9^ Then we consider the utility matrix with $0<\omega_{1}<1$, $0<\omega_{2}<1$ that are pre‐specified, as shown in Table 2.

**TABLE 2:** Utility matrix with two parameters

	E=0	E=1
T=0	$\omega_{1}$	1
T=1	0	$\omega_{2}$

We define the Utility function Ū(p(d,x),ω) as the expectation of the utility matrix in Table 2,

(1) $$\begin{array}{ll} Ū(p(d,x),\omega ) & =\omega_{1}p_{00}(d,x)+p_{10}(d,x)+\omega_{2}p_{11}(d,x)\\ & =\omega_{1}+(1-\omega_{1})p_{E}(d,x)-\omega_{1}p_{T}(d,x)+(\omega_{1}+\omega_{2}-1)p_{11}(d,x). \end{array}$$

When $\omega_{1}+\omega_{2}=1$, the gain in utility when moving from E=0 to E=1 is the same for both levels of T ($1-\omega_{1}=\omega_{2}$), and $Ū(p(d,x)=\omega_{1}+(1-\omega_{1})p_{E}(d,x)-\omega_{1}p_{T}(d,x)$, which yields equivalent solution to $U(p(d,x),\theta )=p_{E}(d,x)-\theta p_{T}(d,x)$. For this special case the models for E & T can be built separately,^5^ and no assumption concerning independence or dependence of E and T is needed. However, when $\omega_{1}+\omega_{2}\neq 1$, this is not the case and the utility function does depend on the correlation between efficacy and toxicity.

For a subject with covariates x, we can directly calculate the optimal dose when the true probabilities p(d,x) are known, by maximizing the utility function across possible dose values. That is

(2) $$d_{opt}(x)=\underset{d}{\text{argmax}}\;Ū(p(d,x),\omega ).$$

### 2.2 Modified utility functions

In many settings including oncology, both efficacy and toxicity increase with increasing dose and clinicians may be unwilling to give treatments associated with high rates of toxicity, even if they are also highly efficacious. To address this concern in dose selection, we can modify the utility function by increasing the penalty on higher values of $p_{T}(d,x)$. As an illustration we will modify the utility function such that if $p_{T}(d,x)\geq 0.3$, we subtract $2\omega_{1}p_{T}(d,x)$ from the utility function, which corresponds to a tripling of the weight for toxicity higher than 0.3 and gives a utility function of

(3) $${Ū}_{1}(p(d,x),\omega )=Ū(p(d,x),\omega )-2\omega_{1}p_{T}(d,x)𝕀[p_{T}(d,x)\geq 0.3].$$

In some settings, it may be desirable to limit the between patient variance in selected optimal dose and/or to avoid extreme doses. This can be accommodated by adding penalty for extreme doses to the utility function. Let $d_{\text{fix}}$ be a population fixed dose that could be pre‐specified as a standard value, or estimated by fitting a dose‐only model without covariates x in it. Then the modified utility function is

(4) $${Ū}_{2}(p(d,x),\omega )=Ū(p(d,x),\omega )-\delta {\left(d-d_{\text{fix}}\right)}^{2},$$

where δ is the penalty parameter and $d_{\text{fix}}$ is the population fixed dose.

**FIGURE 1:**
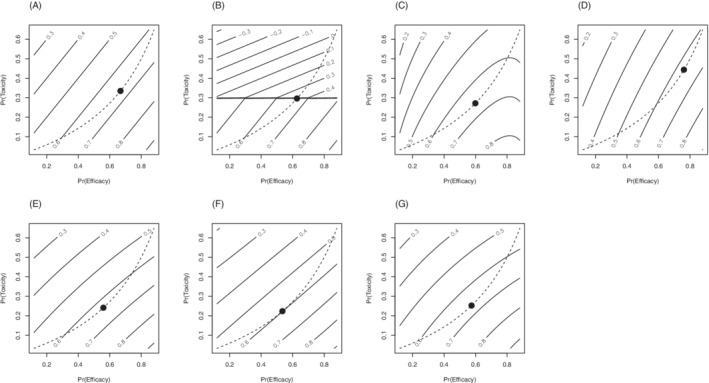
Comparison of utility function contours. (A) Ū with $\omega_{1}=\omega_{2}=0.5$, (B) ${Ū}_{1}$ with $\omega_{1}=\omega_{2}=0.5$, (C) ${Ū}_{2}$ with $\omega_{1}=\omega_{2}=0.5$, δ=0.1, (D) Ū with $\omega_{1}=0.3,\omega_{2}=0.5$ and independent E and T, (E) Ū with $\omega_{1}=0.5,\omega_{2}=0.3$ and independent E and T, (F) Ū with $\omega_{1}=0.5,\omega_{2}=0.3$ and cor(E,T)=0.8, (G) Ū with $\omega_{1}=0.5,\omega_{2}=0.3$ and cor(E,T)=‐0.8

Figure 1 shows the trade‐off contours over the two‐dimensional space with different utility functions. The pairs of ($p_{E},p_{T}$) on each contour are considered to be equally desirable (ie, have the same utility function value), as denoted by the solid lines. For a given patient with covariates x, the $p_{E}(d,x)$ and $p_{E}(d,x)$ among the possible dose range is shown as a dashed line, and the dose that maximize the utility function is selected, as shown by the solid dot. When $\omega_{1}+\omega_{2}=1$, the utility function is a weighted difference of $p_{E}$ and $p_{T}$ and results in straight line contours. The other utility functions have curved or piecewise linear contours. For ${Ū}_{1}$, the utility function decreases rapidly when $p_{T}(d,x)$ is above the pre‐specified toxicity limit of 0.3. ${Ū}_{2}$ has $l_{2}$‐penalty for dose and when the dose is too high, the contours could have negative slopes. When $\omega_{1}+\omega_{2}\neq 1$, the four plots (d) to (g) indicate the influence of different $\omega_{1},\omega_{2}$ values and the correlations of E and T. The lines are curved with $p_{11}(d,x)$, and the joint distribution of E and T plays a role. These plots shows different choices of optimal dose as the utility function changes. For (e) to (g), the correlations are 0, 0.8, and −0.8 with the same set of $\omega_{1},\omega_{2}$ and marginal probabilities for a given patient, the utilities differs a lot, but the optimal doses are similar.

### 2.3 Posterior distribution of utility function

Given the data ${𝒟}_{N}$, the point estimate of the probabilities for the possible doses, denoted by $\hat{p}(d,x)$, can be plugged into Equation (2). Then the individual optimal dose for subject with covariates x is the dose that maximizes the weighted sum of the utility values using the joint probability estimates. When using Bayesian estimation, we will get the posterior distribution of the joint probabilities p(d,x) for the possible doses, and thus the posterior distribution of Ū(p(d,x),ω). The optimal dose for subject with covariates x is selected as the dose that maximize the posterior mean of Ū(p(d,x),ω) given the data ${𝒟}_{N}$.

(5) $$d_{opt}(x)=\underset{d}{\text{argmax}}\;E(Ū(p(d,x),\omega )|{𝒟}_{N}).$$

To assess the uncertainty of the estimation, we can consider the posterior distribution of the utility function. Then an alternative way to define the optimal dose for subject with covariates x is as the dose that maximizes the posterior probability that the utility function is higher than the utility function at some fixed dose which is the same for everyone. With this approach, doses associated with small increases in expected utility will not be selected if they also greatly increase the variance of the expected utility.

(6) $$d_{opt}(x)=\underset{d}{\text{argmax}}\;P(Ū(p(d,x),\omega )>Ū(p(d_{\text{fix}},x),\omega )|{𝒟}_{N}).$$

## 3 MODEL BUILDING

There are various approaches to model the joint distribution of E and T of which we consider three: model the marginal distributions for E and T and then assume independence, or use a copula to link them as the joint distribution, or directly model the 4 level categorical outcome (E,T) on d and x. Instead of using parametric models, we will consider using non‐parametric approaches (eg, random forest) and kernel machines (eg, Gaussian process) to model the joint distribution of E and T.

### 3.1 Random forest

Random forest (RF) for classification constructs multiple trees using bootstrapped samples from the original data. The subject left out in the construction of the kth tree will go down the kth tree to get a classification, which is the out‐of‐bag (oob) classification. Then the proportion of classifications of each type across all the oob classifications for this subject will be used to calculate the predicted probability. First, we use RF to build models for E and T separately to obtain estimates of ${\hat{p}}_{E}(d,x)$ and ${\hat{p}}_{T}(d,x)$ for each subject. After obtaining estimated marginal probabilities, if we assume conditional independence of efficacy and toxicity, then the joint distribution of (E,T) is given by the product of the marginals. Alternatively, we can use a copula to link the marginals as described in the Appendix A. We can plug the ${\hat{p}}_{E}(d,x)$ and ${\hat{p}}_{T}(d,x)$ for each subject into the copula function and maximize the loglikelihood across the dataset ${𝒟}_{N}$ to estimate $\hat{\alpha }$, the correlation parameter in the copula. *Rborist* by Seligman was used to implement the random forest due to its computational efficiency.^10^

In random forest, the predicted probabilities of E or T may decrease over some range of dose for some patients. To prevent this, we can apply constraints during the estimation of the random forest. Specifically, whenever a split on dose is selected, we require the left node (lower dose) to be associated with lower probabilities of E or T than the right node (larger dose).^10^

Instead of modeling marginals and linking them to obtain the joint distribution, we can model the joint probabilities directly. When doing this using methods such as random forests, we can't add monotonicity directly in the model since the desired monotonicity is in terms of each marginal and not in the four joint probabilities $p_{00},p_{10},p_{01},p_{11}$. Thus in this setting we use a post estimation procedure in which we adjust ${\hat{p}}_{00},{\hat{p}}_{10},{\hat{p}}_{01},{\hat{p}}_{11}$ to obtain ${\hat{p}}_{00}^{*},{\hat{p}}_{10}^{*},{\hat{p}}_{01}^{*},{\hat{p}}_{11}^{*}$ so that the corresponding $p_{E}$ and $p_{T}$ are monotone in d for all x. Specifically, we use the Pool‐Adjacent‐Violators Algorithm (PAVA) isotonic transformation for the marginal distribution,^11^ then we will find a set (${\hat{p}}_{00}^{*},{\hat{p}}_{10}^{*},{\hat{p}}_{01}^{*},{\hat{p}}_{11}^{*}$ ) which is close to (${\hat{p}}_{00},{\hat{p}}_{10},{\hat{p}}_{01},{\hat{p}}_{11}$) and also satisfies the monotonicity constraint.

For every patient, using the model of categorical outcomes built on the dataset, we have predictions of (${\hat{p}}_{00},{\hat{p}}_{10},{\hat{p}}_{01},{\hat{p}}_{11}$) for all the dose levels and thus ${\hat{p}}_{E}$ and ${\hat{p}}_{T}$. Then we apply PAVA on ${\hat{p}}_{E}$ and ${\hat{p}}_{T}$ to get ${\hat{p}}_{E}^{*}$ and ${\hat{p}}_{T}^{*}$ which is non‐decreasing in dose across the feasible dose range. At each dose, the joint probability ${\hat{p}}_{11}^{*}$ has a range of $\left[\max(0,{\hat{p}}_{E}^{*}+{\hat{p}}_{T}^{*}-1),\min({\hat{p}}_{E}^{*},{\hat{p}}_{T}^{*})\right]$. One way to perform the adjustment is to minimize $\left|{\hat{p}}_{11}^{*}-{\hat{p}}_{11}\right|$ within the allowed range to get ${\hat{p}}_{11}^{*}$, then ${\hat{p}}_{10}^{*},{\hat{p}}_{01}^{*},{\hat{p}}_{00}^{*}$ can be calculated sequentially from the known ${\hat{p}}_{11}^{*},{\hat{p}}_{E}^{*},{\hat{p}}_{T}^{*}$. Another way is to minimize $\left|{\hat{p}}_{11}^{*}-{\hat{p}}_{11}|+|{\hat{p}}_{10}^{*}-{\hat{p}}_{10}|+|{\hat{p}}_{01}^{*}-{\hat{p}}_{01}|+|{\hat{p}}_{00}^{*}-{\hat{p}}_{00}\right|$, which involves all the joint probabilities.

### 3.2 Gaussian process

As a second approach to estimating the marginal distributions, we use a Gaussian process (GP) approach. Specifically, we model the outcome y as a distorted version of the process f, where f is a non‐linear latent function which has a Gaussian distribution prior with mean 0 and covariance functions K. For example, a continuous outcome might use a normal distribution around f, while a binary outcome would use a logistic link or probit link with f. With the Gaussian prior

f∼GP(0,K)=p(f),

and likelihood

$$y∼\overset{n}{\underset{i=1}{\prod }}p(y_{i}|f_{i}),$$

the Gaussian posterior of p(f|y) is given by

$$p(f|y)\propto \overset{n}{\underset{i=1}{\prod }}p(y_{i}|f_{i})p(f)=\overset{n}{\underset{i=1}{\prod }}p(y_{i}|f_{i})N(0,K),$$

where K is a kernel function that represents the distance between subjects in the covariate space denoted by z=(d,x). Multiple kernel functions could be used and we use a Gaussian kernel with each element in K defined by

$$k(z_{i},z_{j})=\eta^{2}exp(-\frac{1}{2}\overset{Q+1}{\underset{q=1}{\sum }}\rho_{q}^{2}{\left(z_{i}^{\left(q\right)}-z_{j}^{\left(q\right)}\right)}^{2}),$$

for subjects i and j=1,…,N and dimension q=1,…,Q+1. $\eta^{2}$ is called the signal variance or the magnitude, and $\rho_{q}$'s are the characteristic length‐scales of the input‐space for each dimension of z. This covariance function implements automatic relevance determination (ARD),^12^ since the length‐scale $\rho_{q}$ determines how relevant an input is: if the length‐scale is small, the covariance will become almost independent of that input, effectively removing it from the inference. The ARD can be used in selection of the dimension z related to the outcome, especially with high dimension data or sparse data. We assign priors to $\eta ,\rho_{q}$ and estimate the posterior distribution of them from the data.

In our setting, we assume $f_{E}∼N(0,K_{E}),f_{T}∼N(0,K_{T})$ where $K_{E},K_{T}$ are different Gaussian kernel functions for E, T. For each subject, the marginal probabilities are,

$$\mathrm{Pr}(E=0)=1-p_{E}=\Phi (f_{E}+a),\mathrm{Pr}(T=0)=1-p_{T}=\Phi (f_{T}+b),$$

here we use a probit link to connect f to the outcome. Because f are centered around 0, intercepts a,b are used for the unbalanced outcomes. Then the joint probability can be obtained by the Gaussian copula

$$p_{00}(d,x)=\Phi_{2}[\Phi^{-1}(1-p_{E}),\Phi^{-1}(1-p_{T})|\alpha ]=\Phi_{2}[f_{E}+a,f_{T}+b|\alpha ].$$

Alternatively, when assume independence of efficacy and toxicity,

$$p_{00}(d,x)=\Phi (f_{E}+a)\Phi (f_{T}+b).$$

Then the joint probabilities are

$$\begin{array}{ll} p_{10}(d,x) & =\Phi (f_{T}+b)-p_{00}(d,x),\;\\ p_{01}(d,x) & =\Phi (f_{E}+b)-p_{00}(d,x),\; \end{array}$$

and

$$p_{11}(d,x)=p_{00}(d,x)-\Phi (f_{E}+b)-\Phi (f_{T}+b)+1,$$

respectively.

The outcome (E,T) for each subject follows $Multinomial(p_{00},p_{10},p_{01},p_{11})$, then the posterior is given by

$$p(f_{E},f_{T}|E,T)\propto \overset{n}{\underset{i=1}{\prod }}p_{00,i}^{\left[E_{i}=0,T_{i}=0\right]}p_{10,i}^{\left[E_{i}=1,T_{i}=0\right]}p_{01,i}^{\left[E_{i}=0,T_{i}=1\right]}p_{11,i}^{\left[E_{i}=1,T_{i}=1\right]}N(f_{E}|0,K_{E})N(f_{T}|0,K_{T}).$$

We can use a Bayesian approach to estimate $\left(\eta_{E},\rho_{qE},\eta_{T},\rho_{qT},a,b,\alpha ;q=1,\ldots ,Q+1\right)$ jointly with some prior distribution. *rstan* was used to implement the Bayesian analysis of Gaussian process since it is more efficient and explores complicated posterior distributions better compared to *rjags*.^13^

For Gaussian process, there are no methods to ensure monotonicity of predictions with respect to dose across the entire covariate space. Rather, virtual data points can be included and the derivative of the Gaussian process (with respect to dose) at those points can be encouraged to be non‐negative during estimation.^14^

## 4 SIMULATION STUDIES

### 4.1 Settings and scenarios

To evaluate the performance of the different methods, we conducted a simulation study. We consider 11 scenarios by varying the true parameter coefficients, the functional form (eg, exponential or binary or linear), the number of biomarkers, and the sample size. For each scenario and each method of analysis and definition of optimal dose, we fit the models using the training dataset, and evaluated their performance under the true models which are known only in this simulation setting.

In scenario 1, we generated N=200 observations in a training dataset by simulating 5 i.i.d covariates, $x^{\left(1\right)},\ldots ,x^{\left(5\right)}$ from a standard normal distribution, d from Uniform(−1,1), and binary outcomes E and T from the regression model described below with a copula to link them. The marginal probabilities are defined by models with covariates x, dose d, and dose‐covariate interactions dx

$$p_{E}(d,x)=\Phi (\beta_{0,E}+(x,d,dx)\beta_{E}),p_{T}(d,x)=\Phi (\beta_{0,T}+(x,d,dx)\beta_{T}),$$

and α=0.8 was used in the Gaussian copula to link the two marginal distributions. In scenario 1, $\left(\beta_{0,E},\beta_{E})=(0,0.49,-1.11,0.77,1.51,0,1,0.23,0.61,0,1.69,0.5\right)$, $\left(\beta_{0,T},\beta_{T})=(-1.386,0,1.14,-0.33,0,0,1,0.03,0.6,0,-0.42,1.04\right)$. In generating x's, we also applied the constraints that x must satisfy $1+0.23x_{1}+0.61x_{2}+1.69x_{4}+0.5x_{5}>0$ and $1+0.03x_{1}+0.6x_{2}-0.42x_{4}+1.04x_{5}>0$ to reflect the non‐decreasing dose‐efficacy and dose‐toxicity curves. This excludes up to 40% of initially simulated observations.

The other scenarios considered are shown below in Table 3 . In all scenarios the true model coefficients were chosen so that the proportion of observations with E=1 was about 50%‐70% and with T=1 was about 10%‐30%.

**TABLE 3:** List of scenarios

S0	The true E & T model has only d without covariates x
S1	The true E & T models have x,d,dx
S2	The true E & T models have x,d, no dose‐covariate interactions
S3	S1 with $x_{1},x_{2},x_{3}$ correlated with coefficient 0.6
S4	S1 with 15 noise covariates $x_{6},\ldots ,x_{20}$ added to the data
S5	S1 with 195 noise covariates $x_{6},\ldots ,x_{200}$ added to the data
S6	S1 with sample size = 400
S7	S1 with binary covariates x
S8	The true E & T model has x,d,dx, as well as covariate interactions xx
S9	The true models have $exp(x_{4})$ as the covariate
S10	The true models have $I(x_{4}>0)$ as the covariate

The simulation consist of the following steps iterated 1000 times. (1) Generate a training dataset of the specified size (N=200 or 400). (2) Fit models for efficacy and toxicity on the training data. (3) Generate a validation dataset following the same approach as for the training data. (4) Calculate the estimated optimal dose for each patient in the validation data using the models estimated from the training data and a particular method for selecting optimal dose based on the models. Also calculate the true efficacy and toxicity probabilities for each patient, conditional on the estimated optimal dose, using the true models. (5) Calculate the average and SD of the estimated optimal dose values, average probability of efficacy, average probability of toxicity, average value of the true utility function, and average improvement in utility defined as the fraction of the difference between the expectation of the utility from the true model and the expectation of the utility from using a fixed dose. The metrics in step (5) are then averaged over the 1000 simulations to provide a comparison of the various methods for model building and optimal dose selection across various scenarios.

We consider several variations of random forest modeling. To allow for correlated efficacy and toxicity outcomes we take three approaches consisting of using a copula method to link the marginal models (M2), assuming conditional independence (M3) and also directly modeling the 4 level bi‐variate outcome (M8). We also implement a version of random forest in which the dose effects on efficacy and toxicity are constrained to be non‐negative for all patients and compare these results to unconstrained estimation (M4, M5). When using random forest to model the 4 level categorical outcome, we also assess the impact of a post‐estimation use of a PAVA algorithm to enforce monotonicity (M9).

For the Gaussian process, we compared different priors for $\rho_{q}$ including inverse gamma prior, Laplace prior and horseshoe prior. They have similar performance with low dimension of covariates, but the horseshoe prior is better with high dimension as it allows variable selection. Specifically, $\rho_{q}∼N(0,\lambda_{q}^{2}\tau^{2})$ for q=1,…,Q, where $\lambda_{q}∼cauchy(0,1),\tau ∼cauchy(0,1)$. The global parameter τ pulls all the weights globally towards zero, while the thick half‐Cauchy tails for the local scale $\lambda_{q}$ allow some of the weights to escape the shrinkage. With large τ, all ρ's have diffuse priors with very little shrinkage toward 0, but small τ will shrink all the ρ's to 0. All the other parameters have a standard normal distribution as priors. For Gaussian process, we tried both using copula and assuming independence to link the two marginals. The results were very similar so we only include those corresponding to the independence assumption (M10, M11). Although we did investigate imposing monotonicity constraints in Gaussian process modeling through the use of virtual data points, we did not include these methods in our simulation because they are time consuming and in limited simulations appeared to have little effect on the results.

For comparison, a multinomial logistic regression with 4‐level categorical outcome is built on dose only (M12), and the selected optimal dose is adopted as $d_{\text{fix}}$ in M7 and M10. All the methods considered are summarized in Table 4.

**TABLE 4:** List of methods

	Model building	Optimal dose selection
M1	True model	Ū(p(d,x),ω)
M2	RF on marginals with copula	$Ū(\hat{p}(d,x),\omega )$
M3	RF on marginals with independence	$Ū(\hat{p}(d,x),\omega )$
M4	RF on marginals monotone on d with copula	$Ū(\hat{p}(d,x),\omega )$
M5	RF on marginals monotone on d with independence	$Ū(\hat{p}(d,x),\omega )$
M6	RF on marginals monotone on d with copula	$Ū(\hat{p}(d,x),\omega )-2\omega_{1}{\hat{p}}_{T}(d,x)𝕀[{\hat{p}}_{T}(d,x)\geq 0.3]$
M7	RF on marginals monotone on d with copula	$Ū(\hat{p}(d,x),\omega )-\delta {\left(d-d_{\text{fix}}\right)}^{2}$
M8	RF on categorical outcome	$Ū(\hat{p}(d,x),\omega )$
M9	RF on categorical outcome and PAVA to minimize $\left|{\hat{p}}_{11}^{*}-{\hat{p}}_{11}\right|$	$Ū(\hat{p^{*}}(d,x),\omega )$
M10	GP with independence assumption	$P(Ū(p(d,x),\omega )>Ū(p(d_{\text{fix}},x),\omega )|{𝒟}_{N})$
M11	GP with independence assumption	$E(Ū(p(d,x),\omega )|{𝒟}_{N})$
M12	Fixed dosing: multinomial logistic regression on dose	$Ū(\hat{p}(d),\omega )$

*Note*: RF denotes random forest, GP denotes Gaussian process.

The utility matrix plays an important role in optimal dose selection so for each scenario, we consider three utility matrices. The first places higher utility on the outcome of both efficacy and toxicity compared to neither efficacy nor toxicity ($\omega_{1}=0.3,\omega_{2}=0.5$). The corresponding utility function is $Ū(p(d,x),\omega )=0.3+0.7p_{E}(d,x)-0.3p_{T}(d,x)-0.2p_{11}(d,x)$. The second utility matrix we consider, weights these two possible outcomes equally ($\omega_{1}=\omega_{2}=0.5$). In this setting the correlation plays no role as it cancels out of the utility function, as $Ū(p(d,x),\omega )=0.5+0.5p_{E}(d,x)-0.5p_{T}(d,x)$. In the third utility matrix we consider, the outcome of no efficacy and no toxicity is preferred to the outcome of both ($\omega_{1}=0.5,\omega_{2}=0.3$). The utility function is $Ū(p(d,x),\omega )=0.5+0.5p_{E}(d,x)-0.5p_{T}(d,x)-0.2p_{11}(d,x)$.

### 4.2 Results

**FIGURE 2:**
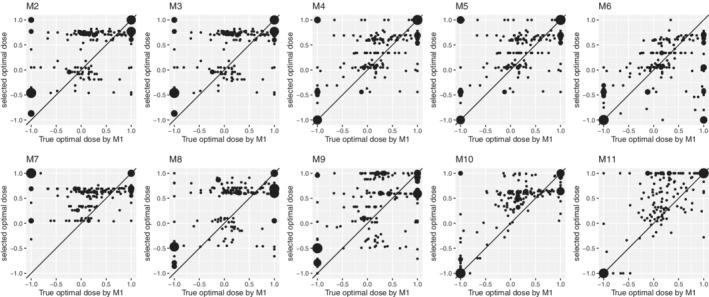
Distribution of optimal dose of n=200 patients for different methods under scenario 1 with utility 1 ( $\omega_{1}=0.3,\omega_{2}=0.5$). The size of the point corresponds to the number of patients

Figure 2 shows the scatter plot of the estimated optimal doses for one representative dataset with n=200 under scenario 1 with $\omega_{1}=0.3,\omega_{2}=0.5$. The size of the point corresponds to the number of patients. Under the true model (M1), the optimal dose would be the minimum or maximum possible value for many patients. Under the fixed dosing model (M12) which ignores covariates, the estimated fixed optimal dose is 0.63 for all the patients in this dataset and is plugged into M7 and M10. The optimal doses selected by different methods are compared with the true optimal dose, as denoted by the diagonal line. For the random forest models on the marginals (M2, M3, M4, M5), the optimal doses for many patients are near the optimal fixed dose. There is little difference between methods using copulas to allow for correlation and assuming independence (M2 vs M3, M4 vs M5), suggesting that use of the copula may affect the estimation of utilities as shown in Figure 1, but does not have a large impact on the distribution of selected doses. As expected, method M6 tends to select lower doses than method M4 due to the individual toxicity limit in the utility function. For method M7, the majority of the patients have optimal doses around the fixed dose because of the dose penalty in the utility function. For the random forest with categorical outcomes (M8, M9), adding PAVA to the predicted probabilities affects the selection of optimal dose. For method M10 which uses Gaussian process and maximizes the probability of utility higher than the fixed dose, the majority of the patients have optimal doses around the fixed dose. For method M11 which uses Gaussian process and maximizes the posterior mean of the utility function, the distribution of the optimal dose is most similar to the distribution from the true model.

Table 5 provides mean values for dose, efficacy, and toxicity across methods. It also provides the average (across simulated datasets) SD of dose and mean values (across simulated datasets) for the average utility (across patients within dataset) under scenario 1. To compare the various methods on a common scale, we calculate the percent of possible increase in expected utility relative to fixed dose, denoted by % IP Ū. Thus by definition the fixed dose approach has 0% improvement and the true models have 100% improvement with other methods generally falling in between these values. When using utility 1, $\omega_{1}<\omega_{2}$, so on average the optimal dose is higher and as a result, average efficacy and average toxicity are both higher. Under utility 3, $\omega_{1}>\omega_{2}$, the average optimal dose is lower, and average efficacy and average toxicity are lower. Under this scenario, within each utility function, use of the true models results in the highest average expected utility, and the fixed dose results in the lowest average of expected utility. The Gaussian process methods (M10, M11) have the highest mean utility values with the other methods having generally similar performance varying somewhat across utility matrices.

**TABLE 5:** Comparison of all methods under scenario 1 with utility 1,2,3 ${}^{a}$

		True	RF		Monotone RF				RF & PAVA		GP		FD
Method		M1	M2	M3	M4	M5	M6	M7	M8	M9	M10	M11	M12
U1	Mean dose	0.221	0.206	0.219	0.233	0.245	−0.017	0.379	0.266	0.28	0.337	0.303	0.464
	SD dose	0.664	0.479	0.473	0.446	0.439	0.478	0.246	0.522	0.513	0.499	0.63	0
	Mean E	0.703	0.623	0.625	0.628	0.629	0.532	0.646	0.649	0.651	0.674	0.687	0.658
	Mean T	0.275	0.294	0.299	0.299	0.304	0.193	0.361	0.322	0.328	0.327	0.325	0.415
	Mean Ū	66.1	60.8	60.8	61	60.9	59.2	60	61.1	61	62.3	**63.1**	58.7
	% IP Ū	100	27.8	27.0	30.0	28.6	5.3	17.0	32.0	30.8	48.4	**59.5**	0
U2	Mean dose	0.017	0.027	0.027	0.03	0.029	−0.117	0.097	−0.009	−0.021	0.033	−0.001	0.142
	SD dose	0.667	0.495	0.495	0.469	0.469	0.483	0.255	0.561	0.539	0.476	0.662	0
	Mean E	0.632	0.571	0.571	0.571	0.57	0.505	0.571	0.568	0.564	0.587	0.6	0.567
	Mean T	0.191	0.223	0.222	0.218	0.218	0.161	0.253	0.213	0.211	0.222	0.208	0.301
	Mean Ū	72.1	67.4	67.4	67.6	67.6	67.2	65.9	67.7	67.6	68.3	**69.6**	63.3
	% IP Ū	100	46.3	46.3	48.4	48.4	43.3	29.4	49.7	48.6	56.2	**70.7**	0
U3	Mean dose	−0.162	−0.095	−0.084	−0.1	−0.086	−0.176	−0.072	−0.178	−0.174	−0.167	−0.215	−0.067
	SD dose	0.646	0.478	0.476	0.455	0.451	0.468	0.253	0.532	0.521	0.389	0.601	0
	Mean E	0.556	0.526	0.529	0.524	0.526	0.489	0.516	0.499	0.499	0.508	0.516	0.5
	Mean T	0.124	0.179	0.184	0.173	0.178	0.146	0.194	0.155	0.157	0.162	0.136	0.229
	Mean Ū	69.6	65.1	65	65.4	65.3	65.5	64.1	65.2	65.2	65.3	**67**	61.6
	% IP Ū	100	43.2	41.9	46.9	45.2	48.4	31.3	44.5	44.1	45.9	**66.8**	0

*Note*: Methods with highest percentage improvement of Utility (%IP Ū) are highlighted in bold.

Abbreviations: FD, fixed dosing; GP, Gaussian process; RF, random forest; True, true model.

${}^{a}$ Utility 1: $\omega_{1}=0.3,\omega_{2}=0.5$; Utility 2: $\omega_{1}=\omega_{2}=0.5$; Utility 3: $\omega_{1}=0.5,\omega_{2}=0.3$.

**FIGURE 3:**
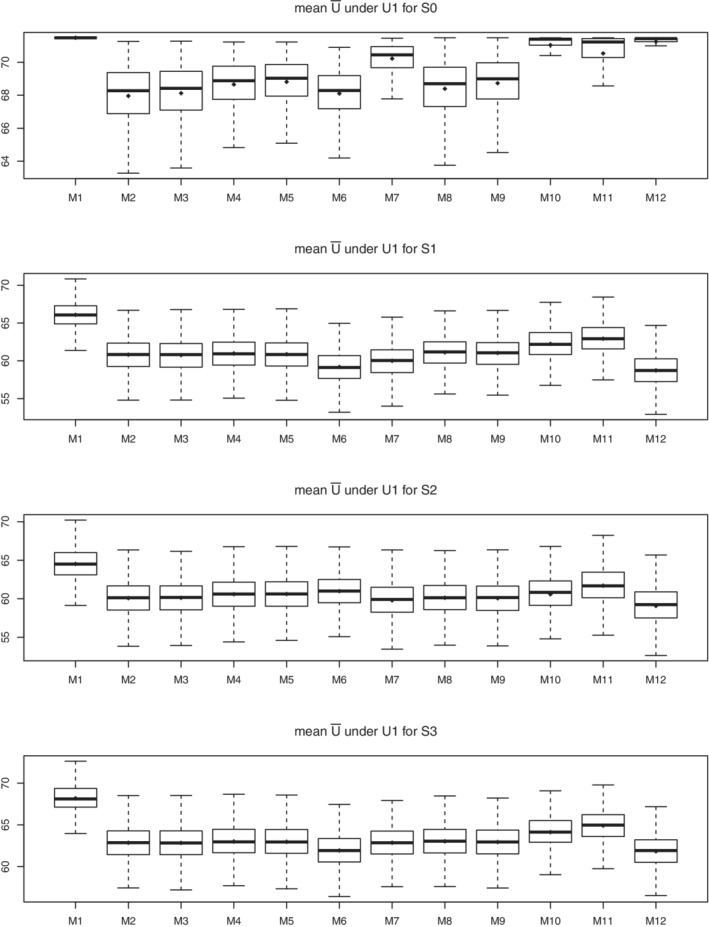
Simulation results for scenario 0, 1, 2, 3 under utility 1 ($\omega_{1}=0.3,\omega_{2}=0.5$). Boxplot of population average of expected utility for 1000 simulation trials

In practice, it is possible that none of the considered covariates (eg, biomarker panel) have any true association with the patient outcomes. To mimic this setting, we also consider a null scenario (S0) in which the true E and T models have only d without covariates x in them. Thus the dose effects are the same across all patients. As shown in Figure 3, the dose‐only model has nearly as high of utility values as are possible using the true model. All other modeling approaches which consider other covariates, generally have poorer utility values than fixed dosing in this setting.

**FIGURE B1:**
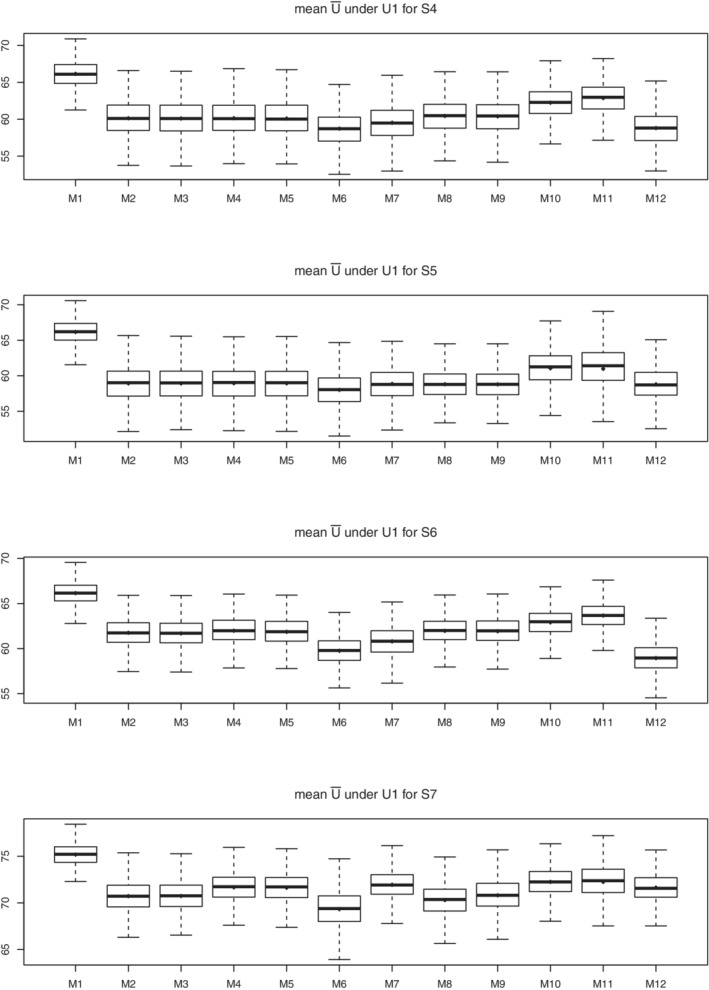
Simulation results for scenario 4‐7 under utility 1. Boxplot of population average of expected utility for 1000 simulation trials

**FIGURE B2:**
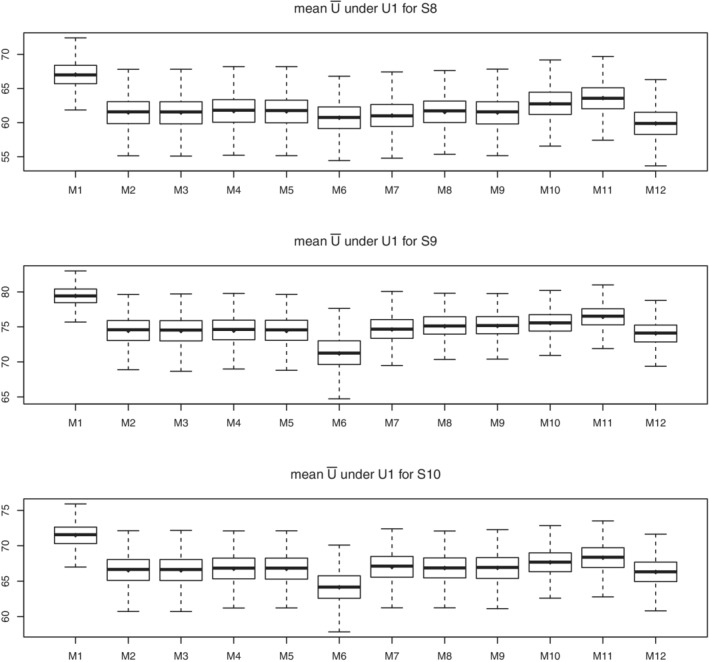
Simulation results for scenario 8‐10 under utility 1. Boxplot of population average of expected utility for 1000 simulation trials

The comparison of expected utility under all scenarios is shown in Figure 3 and figures in the appendix, Figures B1 and B2. Table 6 shows the comparison of percentage of expected utility improvement under all scenarios with utility 1. In scenario 2, when there are only dose main effects and no dose‐covariate interactions, the main effects of the covariates still play a role in optimal dose selection, and the Gaussian process which maximizes the posterior mean of utility function (M11) is better than other methods. In scenario 3, when covariates are correlated, the Gaussian process methods (M11, M10) outperform the other methods. In scenario 4 and 5, with the increased number of noise covariates, the ARD feature of the Gaussian process and the horse‐shoe priors help in model building and methods M12 and M13 have better performance than others, especially in scenario 5 when random forest (M2‐M9) shows no improvement of utility function. In scenario 6, with the larger sample size, the magnitude of the improvement increases, and Gaussian process methods (M10, M11) still perform better than others. In scenario 7 when the covariates are all binary, to our surprise, the random forest methods (M2‐M9) all have poor performance, while Gaussian process methods (M10, M11) still improve the utility function relative to fixed dosing (M12). In scenario 8 to 10, when the marginal models have covariate interactions or are mis‐specified, all the methods have smaller magnitude of improvement, but the Gaussian process methods (M11, M10) still perform better than random forest methods (M2‐M9), showing their robustness. Across all those scenarios, the copula makes no difference compared to assuming independence (M2 vs M3, M4 vs M5). Including the monotonicity in random forest leads to a small improvement in the mean utility function (M4 vs M2). However, adding monotonicity in categorical random forest afterwards does not help, as seen by comparing M9 vs M10. As shown in Figure 2, the optimal dose distribution of Gaussian process which maximizes posterior mean of utility function (M11) is most similar to the true model (M1), and also has the highest percent improvement in utility. Using Gaussian process modeling and selecting dose to maximize the posterior probability of higher utility than associated with fixed dosing (M10), tends to put a lot of patients near the fixed dose, but still improves the mean utility function compared to fixed dose. Besides the above scenarios, we also considered smaller sample sizes and lower efficacy rate of about 20%‐30%, and simulation shows GP maximizing expectation of utility (M11) is still the best (results not shown).

**TABLE 6:** Comparison of percentage of utility function improvement under all scenarios with utility 1 ($\omega_{1}=0.3,\omega_{2}=0.5$)

	True	RF		Monotone RF				RF & PAVA		GP		FD
Scenarios	M1	M2	M3	M4	M5	M6	M7	M8	M9	M10	M11	M12
S1	100	27.8	27.0	30.0	28.6	5.3	17.0	32.0	30.8	48.4	**59.5**	0
S2	100	15.5	16.2	24.7	25.1	32.4	12.3	16.3	15.1	24.7	**47.1**	0
S3	100	14.4	14.2	16.9	16.4	−0.3	15.2	17.5	16.1	35.0	**46.4**	0
S4	100	17.7	17.2	17.8	17.2	−1.9	9.7	22.0	21.3	45.1	**53.6**	0
S5	100	1.4	1.4	1.7	1.6	−11.4	0.8	−0.1	−0.4	**30.8**	29.0	0
S6	100	38.5	37.6	41.7	40.0	10.5	25.5	42.0	41.2	55.0	**65.4**	0
S7	100	−28.4	−28.0	−1.7	−2.8	−68.5	6.4	−40.5	−25.9	**17.1**	16.5	0
S8	100	21.5	21.2	24.2	23.5	10.1	15.5	22.3	21.2	41.1	**52.5**	0
S9	100	6.2	5.7	6.7	5.8	−54.7	10.3	18.5	19.4	26.8	**43.7**	0
S10	100	4.0	3.8	8.3	7.9	−42.9	13.4	10.2	10.7	25.9	**38.4**	0

*Note*: Methods with highest percentage improvement of Utility (%IP Ū) are highlighted in bold.

Abbreviations: FD, fixed dosing; GP, Gaussian process; RF, random forest; True, true model.

### 4.3 Parametric vs non‐parametric models

In the above simulation, we compared different non‐parametric methods and their performance in optimal dose selection. In this section, we compare a subset of the non‐parametric methods with parametric models such as logistic regression (M13), LASSO (M14) and constrained LASSO (CLASSO, M15) proposed by Li et al. All the parametric models are built on dose d, covariates x, and dose‐covariate interactions dx for the marginals and we use copula to link them. We also explore the multinomial logistic regression on 4 categorical outcomes with dose, covariates and interactions (M16), and add PAVA for the non‐decreasing relationship (M17). The results are shown in Table 7. When the parametric models are correctly specified, they are more efficient and give higher utility values than the non‐parametric models, as shown by S1, S4, and S7.

**TABLE 7:** Comparison of percentage of utility function improvement under selected scenarios with utility 1 ($\omega_{1}=0.3,\omega_{2}=0.5$) for selected non‐parametric and parametric methods

	True	RF			GP		FD	Parametric				
Scenarios	M1	M2	M4	M8	M10	M11	M12	M13	M14	M15	M16	M17
S1	100	27.8	30.0	32.0	48.4	59.5	0	75.9	80.4	**84.6**	65.5	63.8
S4	100	17.7	17.8	22.0	45.1	53.6	0	−14.0	67.9	**70.5**	−80.9	−93.7
S7	100	−28.4	−1.7	−40.5	17.1	16.5	0	49.0	**66.3**	65.7	22.1	20.9
S8	100	21.5	24.2	22.3	41.1	52.5	0	62.7	64.2	**68.9**	51.4	52.3
MS1	100	6.3	11.0	7.8	7.1	24.1	0	21.0	25.3	**28.1**	21.3	19.8
MS2	100	30.2	32.7	28.2	22.7	**53.2**	0	27.9	33.1	41.7	−19.9	−20.5
MS3	100	19.8	22.5	19.8	9.5	**32.5**	0	21.9	28.5	30.3	10.5	11.1
MS4	100	8.4	15.2	9.5	8.6	**25.7**	0	1.6	10.7	11.4	−1.0	−0.2

*Note*: Methods with highest percentage improvement of Utility (%IP Ū) are highlighted in bold.

Abbreviations: FD, fixed dosing; GP, Gaussian process; Parametric, parametric models; RF, random forest; True, true model.

Furthermore, we considered more situations when the complexity of the true model increases and the estimation model is mis‐specified. The true efficacy and toxicity models can have more interactions such as covariate interactions $x_{i}x_{j}$ and dose‐covariate‐covariate interactions $dx_{i}x_{j}$. The dose effect is g(d) where g(.) is a non‐linear function. The efficacy model has log(d+1) instead of d, so the efficacy increases faster at lower doses and tends to level off. Similarly, the toxicity model has exp(d) instead of d, so the toxicity increases faster at high doses. In the limited mis‐specified scenarios with detailed description in Table B1, parametric models are quite robust against unspecified interactions as under S8 and MS1, and non‐parametric models have comparable performance. On the other hand, with non‐linear dose effects, non‐parametric models outperform the parametric models as shown by MS2‐4.

## 5 APPLICATION

In this section, we applied the proposed methods to a dataset of liver cancer patients treated with adaptive stereotactic body radiotherapy (SBRT) from 2006 to 2016. The patients come from several clinical trials and observational studies with at least 1 year of potential follow‐up. We utilize the standard measure of toxicity in this setting defined as the occurrence of a 2‐point increase in Child‐Pugh (CP) score within 6 months of treatment. Our primary measure of efficacy is defined as the absence of tumor progression within 1 year of treatment. A total of 188 patients were eligible for inclusion and had the toxicity outcome recorded. Six patients had less than one year of follow‐up for the tumor progression endpoint and were excluded, leaving 182 patients in the final analysis. Overall, 48 patients (26%) had toxicity. A total of 11 (6%) patients had tumor progression within 1 year and are defined as E=0 while 123 patients with no tumor progression are defined as E=1. Patients experiencing the competing risks of death (N=39) or liver transplant (N=9) without prior tumor progression are scored as having no tumor progression (E=1). There were 46 patients who had both efficacy and toxicity observed. The baseline clinical features we considered were pre‐treatment ALBI (Albumin‐Bilirubin) Grade, tumor size GTV, prior liver directed therapy, and pre‐treatment AFP (alpha‐fetoprotein). Patients in this study received tumor doses ranging from 35.7 to 180 Gy, with variation partially due to the clinical trial on which they were enrolled, varying preferences of the treating clinician, as well as patient factors such as cancer stage, tumor location and performance status. In radiation oncology, the dose to the tumor site (most relevant dose for predicting efficacy) is different from the dose received by the normal liver tissue (most relevant for predicting liver toxicity). The ratio of these two doses can be assumed to be constant for an individual patient but varies between patients due to tumor volume and location within the liver. We apply our methods to estimate the optimal tumor dose and calculate the implied normal liver dose for each patient using this fixed ratio. Two patients are missing liver dose and 6 are missing pre‐treatment AFP. Single imputation was used to fill in these missing values using the other covariates and outcomes.

In this setting, achieving efficacy is less important than avoiding toxicity so for the utility matrix, we set $\omega_{1}=0.6$ and $\omega_{2}=0.4$, considering an outcome of neither efficacy and toxicity (E=0,T=0) preferable to an outcome of both toxicity and efficacy (E=1,T=1). For the random forest on the marginal distributions, we include tumor dose, tumor size GTV, and pre‐treatment AFP in the efficacy model, and liver dose, pre‐treatment ALBI, tumor size GTV, and prior liver directed therapy in the toxicity model. For the random forest on categorical outcomes and Gaussian process, we include tumor dose and the ratio of liver dose to tumor dose as well as all clinical features. For the fixed dosing method, we build a multinomial logistic regression model on tumor dose and the ratio of liver dose to tumor dose.

**FIGURE 4:**
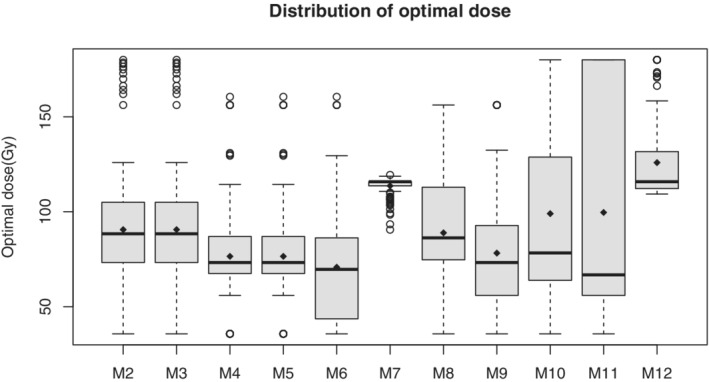
Boxplot of optimal doses by different methods for the 182 patients

Due to variation in the ratio of liver dose to tumor dose, the dose only model (M12) estimates different optimal doses for each patient. It also selects on average the highest individualized optimal tumor dose for each patient, as shown in Figure 4. Similarly, M7 incorporates the dose penalty towards the median dose selected by M12 and exhibits the least variability in selected dose values with a high dose selected for the majority of patients. There is little difference between the independence assumption or using the copula to link the two marginals, as shown by M2 vs M3 and M4 vs M5. As expected, adding the individual level toxicity penalty (M6) lowers the recommended doses. Use of the PAVA monotonicity algorithm on predictions from the random forest on the 4‐category outcomes has some impact on the selected optimal dose (eg, see medians for M8 vs M9). The Gaussian process methods recommend a wide range of doses to different patients, as shown by M10 and M11.

**FIGURE 5:**
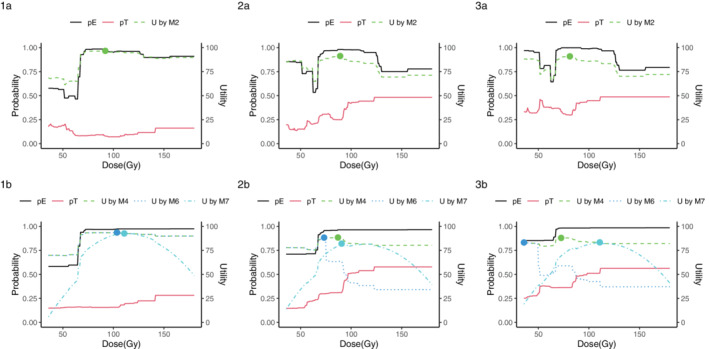
Optimal dose selected by different methods for three patients. Dose‐efficacy and dose‐toxicity curves are denoted by solid lines, expected utility values by different methods are denoted by dashed lines, optimal dose selected by different methods are denoted by points

**FIGURE B3:**
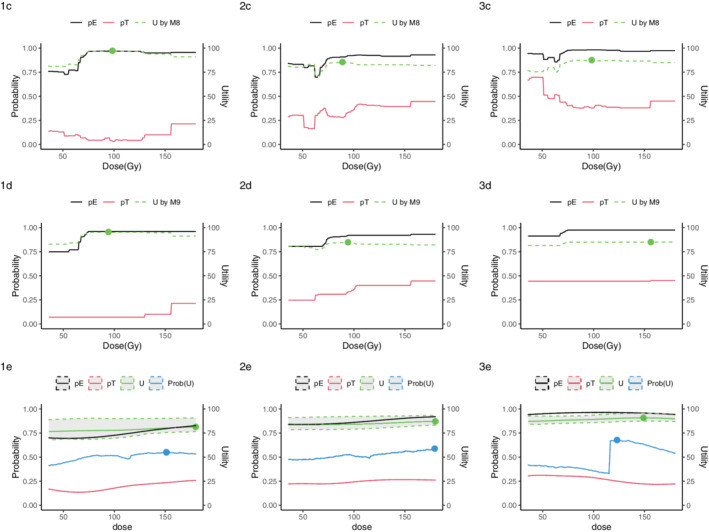
Optimal dose selected by different methods for three patients. Dose‐efficacy and dose‐toxicity curves are denoted by solid lines, expected utility values by different methods are denoted by dashed lines, optimal dose selected by different methods are denoted by points

To illustrate how the proposed utility calculations work we plot in Figure 5 the estimated probabilities of efficacy and toxicity and the corresponding utility functions vs dose for 3 selected patients. In the top row (a) we show the predictions from random forest models for efficacy and toxicity with copula to link them (M2), and the bottom plots (b) show the random forest models for efficacy and toxicity constrained to be monotone in dose and linked with copula (M4). Without forcing monotonicity, each of these 3 patients have regions of dose where, as dose increases, the estimated probability of efficacy decreases, which is not biologically plausible. Imposing monotonicity results in believable estimates and also smooths the dose‐efficacy curves somewhat. The bottom panel (b) of the figure shows optimal doses for methods M4, M6, M7 which all utilize the same random forest models but differ in how they define optimal dose from these models. For patients 1 and 2, imposing monotonicity on the dose efficacy curve, results in a similar optimal dose. Patient 3 represents a patient with high baseline risk of toxicity exceeding what would typically be expected from dose only toxicity models. Because the probability of toxicity is higher than 0.3 even at the lowest dose, method M6 with the individual toxicity penalty recommends the minimal dose. A higher dose is recommended from M7 however, as efficacy increases while toxicity plateaus. Plots showing other methods including M8, M9, M10, M11 on those three patients are included in the Appendix Figure B3. The random forest for the categorical outcome results in non‐monotone dose‐efficacy and dose‐toxicity relationship, and the PAVA which adjust the curves results in a plateau. The Gaussian process have smoother curves for dose‐efficacy and dose‐toxicity. Due to the limited sample size, the credible interval of the estimates is wide as shown by the shadow around the posterior mean of utility.

The random forest method discretizes continuous variables and the prediction curves are not as smooth as standard regression models. The trade‐off however is that in contrast to a parametric model, they can approximate any arbitrary shaped smooth function with sufficient data and number of splits.

## 6 DISCUSSION

We have developed statistical methods to estimate individualized optimal dosing rules using utilities to make the trade‐off between efficacy and toxicity quantitative and explicit via input from clinicians. In addition to quantifying the utility of each possible bivariate outcome, this approach requires models estimating the probability of each possible bivariate outcome as it depends on patient covariates and treatment dose. We have proposed use of flexible machine learning methods such as random forest and Gaussian process to build these models for efficacy and toxicity.

In our simulation studies, whether and how correlation between efficacy and toxicity was modeled, had little impact on the selected dose or on patient outcomes. Constraining the estimated probabilities to be monotone in dose, did provide modest improvements in outcome. Equally important, enforcing monotonicity results in believable results which is important for developing and maintaining trust with non‐statistical collaborators. Monotonicity on a single covariate can be encouraged by using virtual points in the Gaussian process approach,^14^ or as a splitting criteria in random forest and gradient boosting trees. But for BART, current software implementations only allow for monotonicity on all covariates (or none),^15^ which is not appropriate for our setting.

In practice, the utility matrix can be pre‐specified by clinicians either directly or indirectly through a series of questions. Simulations or hypothetical patient data may also be useful to illustrate estimation of optimal dose which may motivate the modification of utility values $\omega_{1}$ and $\omega_{2}$ in further discussion with clinicians. Methods to obtain consensus utilities by summarizing questionnaires from a group of clinicians can also be used, such as the Delphi method.^16^ In future work, individual patient preference for the efficacy and toxicity outcomes can also be considered, to build an individualized utility matrix. One difficulty with such an approach is that it may be difficult for patients to quantify a utility for outcomes they have never experienced. It is also possible to add penalties to the utility function to take into consideration the financial cost of the treatment, which in some situations will depend on dose.

The expectation of the utility matrix Ū(p(d,x),ω) has been used to select the optimal dose for each patient. Given the observed data ${𝒟}_{N}$, frequentist estimation of $\hat{p}(d,x)$ can be plugged into the utility function for optimization. Alternatively, Bayesian estimation will give the posterior distribution of p(d,x). The posterior mean of Ū(p(d,x),ω), which is the average of utility function over the posterior distribution, is used as the common Bayesian approach for optimal dose selection. However, the posterior mean of the utility function ignores the variance of the posterior distribution, which reflects the uncertainty of the estimation. In this article, we also propose to maximize the posterior probability that the utility function is higher than the utility function at a fixed dose. It is possible that the posterior mean of the utility function increases slightly with dose, but also has a larger variance, so there is not much improvement of the posterior probability of an improvement in the utility function. The posterior probability represents how confident we are about the improvement of the utility function, which can help clinicians in decision making. In the simulations, it is shown that using the posterior probability can help to avoid assigning extreme doses to patients, and shrink the individual optimal dose towards the population fixed dose.

We considered a number of different machine learning methods as a component of our procedure, when predictions were required. Non‐parametric machine learning methods are flexible, and make only weak assumptions about the underlying functional form. But they generally require a large sample size to estimate the functions and may suffer from overfitting. Thus the sample size as well as the number of covariates and the expected complexity of the function should play a role in determining which method is appropriate in any setting. With smaller sample size, parametric machine learning methods such as neural networks can be considered as a powerful tool, or classical methods such as logistic regression could be appropriate. Other tree based methods could also be considered, such as BART^17^ and gradient boosting trees.^18^ Using Bayesian estimation, Gaussian process and BART result in smooth predictions and have good performance in terms of AUC and RMSE^19^ compared to random forest and gradient boosting. An additional benefit, is the ability to quantify uncertainty through posterior distributions. The nature of the tree structure in random forest, BART and gradient boosting trees means these methods should be well suited to handle the interaction of dose and covariates. On the other hand, Gaussian process uses a Kernel function to describe the relationship across dose and covariates and should work better with continuous variables such as dose.

The performance of the Gaussian Process method depends critically on the prior distributions. In particular when there are a large number of covariates, we need to select an appropriate prior for ρ, the weight for each covariate in the kernel function. A Laplace prior, which is a Bayesian version of robust Lasso regression, did not perform well in our simulation study when we had a large number of covariates and sparsity. The spike‐and‐slab prior is appealing as it places a substantial point‐mass on zero, but can be computationally intensive with a large number of covariates. The horseshoe prior has been shown to have comparable performance to the spike‐and‐slab prior and is more computationally efficient because of its global parameter and local scales.^20^ We adopted the horseshoe prior in our simulations and found it had good performance, even with a low number of covariates.

There are many opportunities for extending the proposed methods. For example, the outcomes for defining the utility may be more complex than a single binary efficacy and toxicity outcome as considered in this article. Utility values can be assigned to ordinal categorical efficacy and toxicity outcomes and the 2×2 utility matrix can be expanded. Survival outcomes can also be broken down into time intervals and then used to assess the trade‐offs between quality and quantity of life, as in quality adjusted survival analysis.^21^

The setting we consider in this article is where dose can be thought of as continuous, and the data has a large number of distinct dose values. The proposed methods have great potential for application in selecting optimal dose in radiation oncology and also more broadly in oncology to select an optimal dose of chemotherapy or immunotherapy. A continuous dose scheme minimizes information loss, it can also be expanded to settings with sufficient finite and discrete dose levels and may yield similar results,^22^ and the pre‐specified dose level that maximizes the utility can be selected. In other settings there may be a much smaller number of potential doses, say 2 or 3. For this the utility framework is still useful, but different models for the probability of efficacy and toxicity may be preferred.

## CONFLICT OF INTEREST

The authors declare no potential conflict of interests.

## Data Availability

The data that support the findings of this study are available from the corresponding author upon reasonable request.

## Appendix A Using copulas to link two marginal distributions

### A.1

The marginal cumulative distribution function for a binary outcome Y, which could be E or T, is F(0)=Pr(Y≤0|d,x)=1−p,F(1)=Pr(Y≤1|d,x)=1, where p is the probability of Y=1 given d and x.

The copula approach makes use of the property that $\left(V_{E},V_{T})=(F(E),F(T)\right)$ have uniformly distributed marginals. The copula is defined as

$$c(v_{E},v_{T})=\mathrm{Pr}(V_{E}\leq v_{E},V_{T}\leq v_{T})=\mathrm{Pr}(E\leq F^{-1}(v_{E}),T\leq F^{-1}(v_{T})).$$

So for the joint probability given d and x, $\mathrm{Pr}(E=0,T=0|d,x)=\mathrm{Pr}(F(E)\leq F(0),F(T)\leq F(0))=\mathrm{Pr}(F(E)\leq 1-p_{E},F(T)\leq 1-p_{T})$, then

$$p_{00}(d,x)=\mathrm{Pr}(E\leq F^{-1}(1-p_{E}),T\leq F^{-1}(1-p_{T}))=c(1-p_{E},1-p_{T}),$$

$$p_{10}(d,x)=1-p_{T}-c(1-p_{E},1-p_{T}),$$

$$p_{01}(d,x)=1-p_{E}-c(1-p_{E},1-p_{T}),$$

and

$$p_{11}(d,x)=c(1-p_{E},1-p_{T})-p_{E}-p_{T}+1.$$

Then for each subject, the outcome (E,T) is from the multinomial distribution with probabilities $p_{00},p_{10},p_{01},p_{11}$ and the loglikelihood is

$$l=I(E=0,T=0)\log(p_{00})+I(E=1,T=0)\log(p_{10})+I(E=0,T=1)\log(p_{01})$$

$$+I(E=1,T=1)\log(p_{11}).$$

Different copula functions can be used. For this article a Gaussian copula is adopted

$$c(v_{E},v_{T})=\Phi_{2}[\Phi^{-1}(v_{E}),\Phi^{-1}(v_{T})|\alpha ],$$

where α is the correlation parameter of $\Phi^{-1}(v_{E})$ and $\Phi^{-1}(v_{T})$.^23^ The marginal probabilities $p_{E},p_{T}$ can be specified by parametric models and parameters can be estimated jointly in the loglikelihood together with α. Two step estimation methods can also be used,^24^ where in the first step estimates are obtained for the parameters from the marginal models and are then held fixed in the second step, which consists of maximizing the likelihood to estimate the association function parameter. Joe and Xu showed that with the 2‐step estimate for parameters is consistent and asymptotically normally distributed.

## Appendix B Additional results

### B.1

**TABLE B1:** List of scenarios to compare parametric vs non‐parametric models

S1	The true E & T models have x,d,dx
S4	S1 with 15 noise covariates $x_{6},\ldots ,x_{20}$ added to the data
S7	S1 with binary covariates x
S8	The true models have x,xx,d,dx Of the 5 covariates, coefficients are non‐zero for 4 x, 1 xx, 4 dx
MS1	The true models have x,xx,d,dx,dxx Of the 5 covariates, coefficients are non‐zero for 1 x, 1 xx, 1 dx, 2 dxx
MS2	The true models have x,g(d),g(d)x Of the 10 covariates, coefficients are non‐zero for 10 x, 1 g(d)x
MS3	The true models have x,g(d),g(d)x,g(d)xx Of the 5 covariates, coefficients are non‐zero for 1 x, 1 g(d)x, 2 g(d)xx
MS4	The true models have x,xx,g(d),g(d)x,g(d)xx Of the 5 covariates, coefficients are non‐zero for 1 x, 1 xx, 1 g(d)x, 2 g(d)xx
